# SK Channel Modulates Synaptic Plasticity by Tuning CaMKIIα/β Dynamics

**DOI:** 10.3389/fnsyn.2019.00018

**Published:** 2019-10-31

**Authors:** Amita Shrestha, Razia Sultana, Charles C. Lee, Olalekan M. Ogundele

**Affiliations:** Department of Comparative Biomedical Sciences, Louisiana State University School of Veterinary Medicine, Baton Rouge, LA, United States

**Keywords:** NMDAR-GluN1, SK2/3, CaMKIIα, hippocampus, ISI, firing rate

## Abstract

N-Methyl-D-Aspartate Receptor 1 (NMDAR)-linked Ca^++^ current represents a significant percentage of post-synaptic transient that modulates synaptic strength and is pertinent to dendritic spine plasticity. In the hippocampus, Ca^++^ transient produced by glutamatergic ionotropic neurotransmission facilitates Ca^++^-Calmodulin-dependent kinase 2 (CaMKII) Thr286 phosphorylation and promote long-term potentiation (LTP) expression. At CA1 post-synaptic densities, Ca^++^ transients equally activate small conductance (SK2) channel which regulates excitability by suppressing Ca^++^ movement. Here, we demonstrate that upstream attenuation of GluN1 function in the hippocampus led to a decrease in Thr286 CaMKIIα phosphorylation, and increased SK2 expression. Consistent with the loss of GluN1 function, potentiation of SK channel in wild type hippocampus reduced CaMKIIα expression and abrogate synaptic localization of T286 pCaMKIIα. Our results demonstrate that positive modulation of SK channel at hippocampal synapses likely refine GluN1-linked plasticity by tuning dendritic localization of CaMKIIα.

## Introduction

The hippocampus is the center for processing of spatial working memory. At hippocampal synapses, synaptic long-term potentiation (LTP) is dependent on glutamatergic neurotransmission (Cacucci et al., 2007; Allen et al., 2016). Specifically, ionotropic neurotransmission mediated by N-Methyl-D-Aspartate Receptor 1 (NMDAR) underlie the functional basis of synaptic structural plasticity that determines the pattern of neural encoding (Gustin et al., 2011; Tovar et al., 2013; Coultrap et al., 2014; Huang and Gibb, 2014; Ratnadurai-Giridharan et al., 2014; Yao and Zhou, 2017; Yi et al., 2017; Rebollo et al., 2018). The loss of NMDAR function has been implicated in several developmental neurocognitive disorders and may represent a convergence for disease progression. Notably, NMDAR hypofunction has been reported in patients and experimental animals exhibiting behavioral symptoms of autism spectrum disorder, bipolar depression, and schizophrenia (Seillier and Giuffrida, 2009; Uchino et al., 2010; Gustin et al., 2011; Namba et al., 2011; Ramsey et al., 2011; Anticevic et al., 2012; Duffney et al., 2013; Snyder and Gao, 2013; Aow et al., 2015; Stein et al., 2015; Ogundele and Lee, 2018). Owing to its role in synaptic plasticity, loss of NMDAR function often leads to a change in synaptic morphology (González Burgos et al., 2012; Bosch et al., 2014; Chazeau and Giannone, 2016; Bustos et al., 2017; Frangeul et al., 2017; Forrest et al., 2018). To this effect, synaptic perturbations in the cognitive centers translate into impairment of neural encoding of instantaneous (working) memory.

In synaptic potentiation, high-frequency spiking events involve the transient surge of postsynaptic Ca^++^ current that is mediated by NMDAR-linked ionotropic neurotransmission (Xia et al., 1996; Coultrap and Bayer, 2012). In addition to the modulation of synaptic plasticity, the transient Ca^++^ current also couples LTP to cellular regulation and gene expression (Xia et al., 1996; Li et al., 2001; Yao and Wu, 2001; Salter and Kalia, 2004; Yoshii and Constantine-Paton, 2007; Coultrap and Bayer, 2012; Stein et al., 2015; Takei et al., 2015; Sanderson et al., 2016; Wang et al., 2016; Swiatkowski et al., 2017). Ca^++^ surge produced by NMDAR activates synaptic Ca^++^-Calmodulin-dependent kinase 2 (CaMKII) by facilitating autophosphorylation at T286/287 sites of the 9α/3β components of the holoenzyme. Consequently, T286 phosphorylated CaMKIIα targets other synaptic substrates (S831 site of GluA1) and promotes fast-spiking activity that is characteristic of LTP (Lisman et al., 2012; Lai and Ip, 2013; Coultrap et al., 2014; Hell, 2014; Hlushchenko et al., 2016; Khan et al., 2016; Nakahata and Yasuda, 2018). On the other hand, the transient Ca^++^ currents produced by NMDAR ionotropic neurotransmission also activate small conductance channels that are co-localized with NMDAR at postsynaptic densities. Repetitive activation of SK2 attenuates post-synaptic transient Ca^++^ current, and leads to a decline in the frequency of neuronal firing (Lee et al., 2003; Hammond et al., 2006; Lin et al., 2008; Ohtsuki et al., 2012; Ballesteros-Merino et al., 2014; Lee and MacKinnon, 2018).

Activation of NMDAR, and accompanying calcium release, are not isolated events in LTP. Modulation of synaptic potentiation events that underlie LTP involves the movement of monovalent K^+^ ions through the SK2, and constitutes an ion channel gating mechanism (Xia et al., 1998; Stackman et al., 2002; Ngo-Anh et al., 2005; Hammond et al., 2006; Lin et al., 2008). Together, LTP embodies the movement of ions in an organized process of oscillation between Ca^++^ and K^+^ currents. The efflux of K^+^ ions through SK2 decreases synaptic strength by attenuating neuronal firing while creating a state of refractoriness. Positive modulation of SK2 has also been shown to alter frequency-dependent LTP and neural encoding in the hippocampus (Maylie et al., 2004; Lin et al., 2008). Previous studies suggest that SK2 modulate LTP by producing afterhyperpolarization current (I_AHP_) that determines the interspike interval for evoked potentials (Hammond et al., 2006; Lin et al., 2008; Maingret et al., 2008). Additionally, by suppressing transient Ca^++^ current associated with NMDAR, SK2 refine synaptic strength through an activity-linked feedback loop (Ngo-Anh et al., 2005; Kuiper et al., 2012; García-Negredo et al., 2014; Lee and MacKinnon, 2018).

Elsewhere, we reported that the pharmacologically-induced NMDAR hypofunction suppressed CaMKII expression, and increased SK2 expression in the hippocampus of WT mice (Ogundele and Lee, 2018). This outcome suggests that T286 phosphorylation of CaMKIIα and expression of SK2 may be inversely related in NMDAR hypofunction. Here, we tested the hypothesis that SK2 regulation of synaptic excitability may impact CaMKII synaptic localization. We assessed the structural and physiological properties of hippocampal synapses after genetic ablation of GluN1 sub-unit of NMDAR. In addition to dendritic spine perturbations, an upregulated SK2 expression was accompanied by a loss T286/T287 phosphorylation of CaMKII in the hippocampus. Likewise, positive modulation of SK channel in a wild type (WT) hippocampus (normal GluN1) dysregulates T286/T287 phosphorylation, and synaptic localization of CaMKII. Together, our results suggest that SK2 regulate hippocampal excitability—in part—by modulating dendritic localization of CaMKII.

## Materials and Methods

### Animals

Adult male and female animals were acquired from the Jackson’s Lab (Bar Harbor, ME, USA). The animals were bred to generate F_1_ offspring which were later inbred for two generations. All experimental animals were housed under standard laboratory conditions of 12 h alternating light and dark cycle. Food and water were provided *ad libitum*. All animal handling procedures were approved by the Institutional Animal Care and Use Committee of the Louisiana State University School of Veterinary Medicine. The animals used for this study weighed ~24 gm.

### Adeno-Associated Virus (AAV) Injection

Mice were anesthetized by intraperitoneal Ketamine/Xylazine (100 mg/Kg:10 mg/Kg) injection and assessed for pain sensation by toe pinching. The head of the mouse was gently fixed in a multi-rail stereotaxic apparatus (Köpf Instruments, Tujunga, CA, USA) for a craniotomy procedure. The Adeno-associated virus (AAV) cocktail was injected into the anterior hippocampus at coordinates (AP: −1.94 mm, ML: 1.0 mm, DV: 1.5 mm) relative to the Bregma. Approximately 800 nl of AAV cocktail was delivered at the rate of 60 nl per min (3 min interval) using a manual Hamilton’s syringe holder (World Precision Instruments Inc., Sarasota, FL, USA). After the last injection, the syringe was allowed to stay in place for 15 min before it was gradually withdrawn.

### Hypomorphic Mutation of GluN1

Serotype 5 AAVs were used in this study. AAV-CMV-Cre-eGFP (ΔGluN1^Hypomorph^) or control AAV-CMV-eGFP (GluN1^flx/flx^) was injected into the hippocampus of GluN1 floxed mice (Jax: 005246; B6.129S4-Grin1tm2Stl/J). The expression of the reporter protein (eGFP) was verified by fluorescence imaging after 21 days. AAV-CMV-eGFP and AAV-CMV-Cre-eGFP were procured from the University of North Carolina Vector Core.

### Hippocampal Expression of ChR2

Double floxed AAV-EF-1a-ChR2-eYFP-WPRE (University of North Carolina Vector Core) was injected bilaterally into the hippocampus of CaMKII-Cre mice (Jax: 005359; B6.Cg-Tg(Camk2a-cre)T29-1Stl/J) to express light controlled channelrhodopsin (ChR2; *n* = 4). The expression of the ChR2 in dendritic spines was verified by fluorescence expansion microscopy after 21 days (Tillberg et al., 2016; Chang J. B. et al., 2017). In a separate set of *n* = 5 mice, we crossed Ai27D mouse (Jax: 012567; RCL-hChR2(H134R)/tdT-D) with the CaMKII-Cre line. The F1 offspring were inbred to create homozygous Ai27D:CaMKII-Cre mice expressing ChR2 in the hippocampus. Both CaMKII-Cre:ChR2 and Ai27D:ChR2 mice were used for photostimulation experiments (blue light; 470 nm).

### Adult Hippocampal SK Channel Positive Modulation (48 h)

An ICV cannula guide was positioned in the CA1 of a GluN1^flx/flx^ mouse by stereotaxic surgery (AP: −1.94 mm, ML: 1.0 mm), and affixed with a dental cement. The cannula guide was covered with a dummy cannula. Seven days after the implant, the dummy cannula was replaced with an ICV cannula for a single dose (10 μM CyPPA) drug delivery [SK2/3(+)]. The drug solution was delivered at the rate of 10 μL/min using a manual Hamilton’s syringe holder (World Precision Instruments Inc., Sarasota, FL, USA).

### Specimen Preparation

Deeply anesthetized mice were euthanized in an isoflurane chamber. The animals were transcardially perfused with 10 mM PBS (pH 7.4) and the whole brain was harvested. The brain was rapidly placed in cold artificial cerebrospinal fluid (ACSF) maintained on ice, and saturated with 95% O_2_/5%CO_2_. A clean razor blade was used to cut the brain along the sagittal plane. The hippocampus was microdissected and extracted from the right and left hemispheres.

### Immunoblotting

The harvested hippocampal tissue was kept in tubes and stored at −80°C until further processing. Frozen hippocampal tissue was incubated on ice with RIPA lysis buffer containing protease and phosphatase inhibitor cocktail. After 30 min, the incubated tissue was rapidly homogenized to obtain tissue lysate. The homogenate was centrifuged to obtain supernatants containing cytoplasmic, membrane, and synaptic fragments. Hippocampal lysate (10 μl) containing 10 μg of protein was processed for SDS-PAGE electrophoresis. After western blotting (wet transfer), Polyvinylidene fluoride membrane (PVDF) was incubated in Tris-buffered saline with 0.01% Tween 20 (TBST) for 15 min (TBST) at room temperature. Afterward, the membrane was blocked in 3% bovine serum albumin (prepared in TBST) for 50 min at room temperature. The protein of interest and housekeeping protein were detected using the following primary antibodies; Rabbit anti KCNN2 Antibody (ThermoFisher Scientific #PA5-41071); Mouse anti CaMKIIα Antibody (ThermoFisher Scientific #MA1-048), Rabbit anti Phospho-CaMKIIα/β:T286/T287 (Cell Signaling #12716), Rabbit anti Phospho-CaMKII T305/306 (ThermoFisher Scientific #702357), Rabbit anti-Phospho-CaMKIIβ/δ/γ:T287(ThermoFisher Scientific #PA5-37833), and Rabbit anti NMDAR1:NR1 Polyclonal Antibody (ThermoFisher Scientific #PA3-102). All primary antibodies were diluted in the blocking solution at 1:1,000. Subsequently, the primary antibodies were detected using Chicken anti-Rabbit-HRP (ThermoFisher Scientific #A15987; 1:5,000) or Donkey anti-Mouse-HRP (ThermoFisher Scientific #A16017; 1:5,000) secondary antibody. The reaction was developed using a chemiluminescence substrate (ThermoFisher-#34579). In order to normalize protein expression, the membranes were treated with Restore PLUS Western Blot Stripping Buffer (ThermoFisher Scientific #46430), and re-probed with β-Actin (8H10D10) Mouse mAb HRP Conjugate (Cell Signaling #12262S). Protein expression (SK2, GluN1, CaMKII) was normalized per lane using the corresponding β-Actin expression. However, for phosphorylated CaMKII (T286, T287, and T305/306 pCaMKII), normalization was done with the base protein expression (CaMKII).

### Slice Preparation and Acute Brain Slice Treatment

For *ex vivo* acute treatment, the hippocampus was microdissected (bilateral) and incubated in oxygenated ACSF with 95% O_2_/5%CO_2_ constantly being bubbled through the ACSF (ACSF; in mM 125 NaCl, 25 NaHCO_3_, 3 KCl, 1.25 NaH_2_PO_4_, 1 MgCl_2_, 2 CaCl_2_ and 25 Glucose). The set up was maintained on a water bath at 37°C. At the onset of the experiment, 10 μM L-Glutamate was added to the ACSF to induce synaptic activation. After 10 min, 10 μM autocamtide-related inhibitory peptide (A2RIP; a CaMKII inhibitor; Li et al., 2017) and 10 μM CyPPA (concentration-dependent SK2 potentiator; Kasumu et al., 2012) was added to the incubation bath. The set up was maintained for 1 h and the hippocampus was processed to isolate synaptosomal and shaft-cytosol extract using the sucrose gradient method (Supplementary Figure S1), followed by immunoblot validation (Supplementary Figure S2). Gel electrophoresis and western blotting were performed as described above for whole lysates. Here, we used the following primary antibodies—Rabbit anti CaMKIIα Antibody (Cell Signaling #11945S), Rabbit anti Phospho-CaMKIIα/β:T286/T287 (Cell Signaling #12716), Rabbit anti phospho-CaMKII T305/306 (ThermoFisher Scientific #702357), and Rabbit anti-Phospho-CaMKIIβ:T287(ThermoFisher Scientific #PA5-37833)-to detect CaMKII and phosphorylated isoforms (T286, T287 and T395/306) in synaptosomal and shaft-cytosol extracts. Effective separation of cellular component was verified by GluN1 (ThermoFisher Scientific #PA3-102), PSD-95 (Cell Signaling #3450S), ErK1/2 (ThermoFisher Scientific #PA1-4703) and pErK1/2 (ThermoFisher Scientific ABfinity^TM^ Antibody #700012) localization in synaptosomal and shaft-cytosol extracts. A reciprocal test for an effective separation was done by verifying the absence of PSD-95/GluN1 and ErK1/2/pErK1/2 in cytosolic and synaptosomal extracts, respectively.

### Immunofluorescence

After transcardial perfusion with PBS, the animal was perfused with 4% phosphate-buffered paraformaldehyde (PB-PFA). The whole brain was removed and fixed overnight in 4% PB-PFA. Subsequently, the whole brain was transferred into freshly prepared 4% PB-PFA containing 30% sucrose for cryopreservation for at least 72 h. Free-floating cryostat sections (40 μm) were obtained and preserved in 48-well plates containing 10 mM PBS at 4°C. The sections were washed three times (5 min each) in 10 mM PBS (pH 7.4) on a slow orbital shaker (35 rpm). Blocking was performed in 5% Normal Goat serum (Vector Labs #S-1000), prepared in 10 mM PBS + 0.03% Triton-X 100, for 1 h at room temperature. The sections were incubated overnight at 4°C in Rabbit anti SK2 Antibody (ThermoFisher Scientific #PA5-41071) and Rabbit anti α-actinin Antibody (ThermoFisher #701914). The primary antibodies were diluted in blocking solution (10 mM PBS + 0.03% Triton-X 100 and 5% normal goat serum). After primary antibody incubation, the sections were washed twice in 10 mM PBS (5 min each), and labeled with a secondary antibody—Goat anti-Rabbit Alexa 568 (ThermoFisher Scientific #A-11036)—diluted in the blocking solution. Secondary antibody incubation was done for 1 h at room temperature, with gentle shaking. Immunolabeled sections were washed and mounted on gelatin-coated slides using ProLong^TM^ Diamond Antifade Mountant (ThermoFisher Scientific #P36970).

### Microscopy and Quantification

Fluorescence images were acquired in a Nikon-*NiU* fluorescence upright microscope that is configured for 3D imaging. Z-stacks were obtained across a depth of 15 μm and rendered as 3D (eGFP) or converted into 2D images through the “extended depth of focus” option in Nikon Element Advanced Research software. Normalized fluorescence intensity for immunolabeled proteins in the hippocampus was determined in optical slices for serial section images (*n* = 5 per mouse brain). Cell counting and fluorescence intensity quantification were done using Nikon Element AR and ImageJ software. Average count and intensity were determined per field for *n* = 12 fields of view.

### Spine Morphology

#### Fluorescence

Vibrotome sliced 100 μm thick sections containing the hippocampus were prepared in oxygenated aCSF and rapidly fixed in 4% PFA. DIL Neurotracer paste (ThermoFisher scientific #N22880) was applied to the hippocampus with the tip of a pulled capillary glass tube. After DIL treatment, the sections were incubated in a PBS humidified chamber overnight at room temperature. Subsequently, the sections were washed in PBS and blocked with 5% normal goat serum prepared in PBST. After blocking, sections were incubated overnight in Rabbit anti-Mouse Synaptophysin antibody (ThermoFisher Scientific #MA5-14532) prepared in the blocking serum solution. The morphology of dendritic spines with synaptophysin expression was imaged in an Olympus FluoView10i Confocal laser scanner. Subsequent quantification of dendritic spines was done in ImageJ.

#### Transmission Electron Microscopy (TEM)

Microdissected hippocampal tissue was fixed in primary EM fixative composed of 1.6% paraformaldehyde, 2.5% glutaraldehyde, and 0.03% CaCl_2_ in 0.05M cacodylate buffer (pH 7.4). The hippocampus was trimmed into ~1 mm sections using a sharp razor and transferred into a fresh fixative for 2 h at room temperature. In subsequent steps, the samples were washed in 0.1 M cacodylate buffer supplemented with 5% sucrose and fixed in 2% osmium tetroxide for 1 h at RT. The sections were washed in water, then in-block stained with 2% uranyl acetate prepared in 0.2 M sodium acetate buffer (pH 3.5), for 2 h. Stained sections were dehydrated in ascending grades of alcohol and propylene oxide. The processed sections were embedded in Epon-Araldite mixture and polymerized for 24 h at 60°C. Tissue blocks were sectioned using a Leica Ultratome (Leica EM UC7). Thin (80 nm) sections were recovered and stained with lead citrate for 5 min. Transmission electron photomicrographs were obtained in a JEOL 1400 Transmission Electron Microscopy (TEM) microscope, equipped with a GATAN digital camera. All reagents for electron microscopy were from EMS (Hatfield, PA, USA). A typical synapse was identified by the presence of synaptic vesicles, postsynaptic mitochondria, and post-synaptic density. The length of dendritic spines was determined in TEM photomicrographs. As such, we estimated the distance between the post-synaptic density, and the stalk of the dendritic spine.

#### Acute Extracellular Recording

*In vivo* recording was carried out in the hippocampus of anesthetized mice. Animals were deeply anesthetized with Urethane (0.2 mg/Kg i.p.), then the head was affixed on a stereotaxic frame. For combined recording and photostimulation, CaMKII-Cre::ChR2 mice were anesthetized with ketamine/xylazine. Mice were tested for toe pinch response to ensure the absence of sensation before the commencement of the procedure. A craniotomy was done in order to expose the dura. Drops of ACSF was applied to this area to prevent dryness. Under a digital dissection microscope, the dura over the exposed brain area was carefully excised using a bent needle tip. An acute neural probe, with a 10 mm long and 50 μm thick shank was used for this procedure (Neuronexus, Ann Arbor, MI, USA). The probe shank carried four electrodes arranged as a tetrode, with an inter-electrode distance of 25 μm. The electrodes were connected to a pre-amplifier head stage (Intantech, Los Angeles, CA, USA), tethered to a recording controller and amplifier system (Intantech, Los Angeles, CA, USA). The electrode was gently lowered into the brain tissue using an ultrafine (μm range) hydraulic micromanipulator (Narishige, Japan) to reach the CA1 dendritic field (radiatum/molecular layer) at stereotaxic coordinates (AP: 1.94 mm, ML: 1.0 mm, DV: 1.5 mm) relative to the Bregma. Stainless steel ground wires soldered onto the head stage-electrode adapter (Neuronexus; A4 to Omnetics CM32 adapter) were tied to a ground screw that was fixed in the occipital bone. For recording procedure involving optogenetics, the recording electrode shank, optic fiber and Hamilton’s syringe were stereotactically positioned in the CA1. TTL (Prizmatix, Southfield, MI, USA) driven 470 nm LED source (Thorlabs, Newton, NJ, USA) was triggered to generate 50 ms pulses over a 789 s duration (at 1 Hz). In three separate groups, we applied: (1) photostimulation (470 nm); (2) photostimulation with 100 nM Apamin (SK2 blocker; Kasumu et al., 2012) infusion; and (3) photostimulation combined with 10 μM CyPPA (SK2 positive modulation) infusion.

The stereotaxic apparatus, micromanipulator, electrode and subject mouse were kept in a Faraday cage and connected to the amplifier ground. At the onset of each recording procedure, the impedance of the electrodes was determined at 1 KHz. For all recording sessions in this study, impedance measurement for the silicon tetrodes ranged between 0.6 and 3.1 Ω. Single unit activity was recorded by setting the cut-off frequency as 250 Hz and 7.5 KHz, respectively for lower and upper limits, sampled at 20 KHz/s. Neural activity was monitored for 20 min to ensure the stability of the animal’s vitals before the commencement of recordings. Continuously recorded spike trains from the CA1 dendritic field was processed in an Offline Spike Sorting software (*OFSS*; Version 4; Plexon Inc., Dallas, TX, USA). Further analysis of the sorted spikes was done in Neuroexplorer Version 5 (Nex Technologies, Colorado Springs, CO, USA).

#### Neural Spike Processing and Analysis

Neural spikes were extracted from the continuous data through threshold crossing in the OFSS. The extracted spikes were sorted into clusters using a combination of Valley seeking and K-means clustering methods. Spikes were assigned to single unit clusters through a 3-dimensional space principal component analysis (PCA) projection. Where necessary, unsorted spikes were assigned to clustered units, or invalidated if outlying. Sorted neural spike waveforms, clustered units, and up-sampled continuous data were exported into the Neuroexplorer software for analysis of spike properties.

#### Social Interaction Test

Mice were habituated in the testing area for 24 h before the commencement of the behavioral test (Kaidanovich-Beilin et al., 2011). At each phase of the test, the compartment was wiped with 70% isopropyl alcohol to prevent odor-specific cues and bias in the subsequent steps. Two smaller holding compartments in the testing area were designated “E (Empty)” during the habituation trial (5 min). For the sociability test, a stranger mouse (S1_0_; stranger 1) was introduced into one of the compartments while the subject animal was re-introduced into the chamber (5 min). After an inter-trial time of 30 min, we introduced a second stranger mouse (S2), along with the first stranger (now S1_n_) into two separate holdings (social novelty test). The contact with (E or S1_0_), and (S1_n_, S2) were estimated to determine sociability [S1_0_/(E+S1_0_)] and social novelty [S2/(S2+S1_n_)] indices respectively. While “0” represents the first encounter with S1 during sociability test, “*n*” represents the second encounter with S1 in a novel position during the social novelty test. Therefore, different time measurements were done at S1_0_ and S1_n_. The time spent in contact with E, S2, S1_0,_ and S1_n_ in the sociability and social novelty tests were estimated blindly by an independent investigator using a software—Ethovision (Noldus, Leesburg, VA, USA).

#### Statistics and Sample Size

Outcomes for WT (*n* = 4), GluN1^flx/flx^ (*n* = 4), ΔGluN1 (*n* = 7) were compared in One-Way analysis of variance (ANOVA) with Tukey *post hoc* test. Statistical comparison for GluN1^flx/flx^ (*n* = 4) vs. ΔGluN1 (*n* = 4) or Control (*n* = 4) vs. SK2/3(+; *n* = 4) were done in *T*-test analysis. For ACSF (*n* = 4), L-Glut (*n* = 4), L-Glut+A2RIP (*n* = 4), and L-Glut+CyPPA (*n* = 4), outcomes were compared in One-Way ANOVA with Tukey *post hoc* test. For photostimulation electrophysiological recording experiments involving CaMKII-Cre::ChR2, *n* = 3 mice were used per group (light OFF, light ON, light ON+Apamin, and light ON+CyPPA). Statistical analysis was performed in One-Way ANOVA with Tukey *post hoc* test. All statistical analysis was done in GraphPad Prism Version 8. Results are presented as bar graphs with error bar depicting the mean and standard error of mean, respectively. Also, as pie charts where applicable.

## Results

### Hypomorphic Attenuation of GluN1 Function

**Figure 1:**
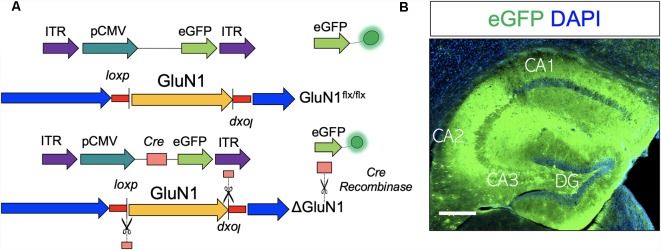
Adeno-associated virus (AAV)-mediated ablation of GluN1 in the hippocampus. **(A)** Schematic illustration of AAV-CMV-eGFP and AAV-CMV-Cre-eGFP construct. **(B)** AAV expression is verified by eGFP (reporter) fluorescence in the hippocampus. Scale bar = 100 μm.

In order to create GluN1 hypomorphic mutation (ΔGluN1^hypomorph^; ΔGluN1 used interchangeably), AAV (Figure 1A) that expresses *Cre* recombinase (AAV-CMV-Cre-eGFP) or a control AAV (AAV-CMV-eGFP) was stereotactically injected into the anterior hippocampus of GluN1 (Grin1) floxed mice. Three weeks after hippocampal AAV injection, transfection was verified by fluorescence imaging of the reporter (gene) protein harbored by the AAV constructs (eGFP). As shown in Figure 1B, hippocampus transfected with AAV produced eGFP fluorescence after 3 weeks.

### Dendritic Spine Morphology and Synaptic Structure

**Figure 2:**
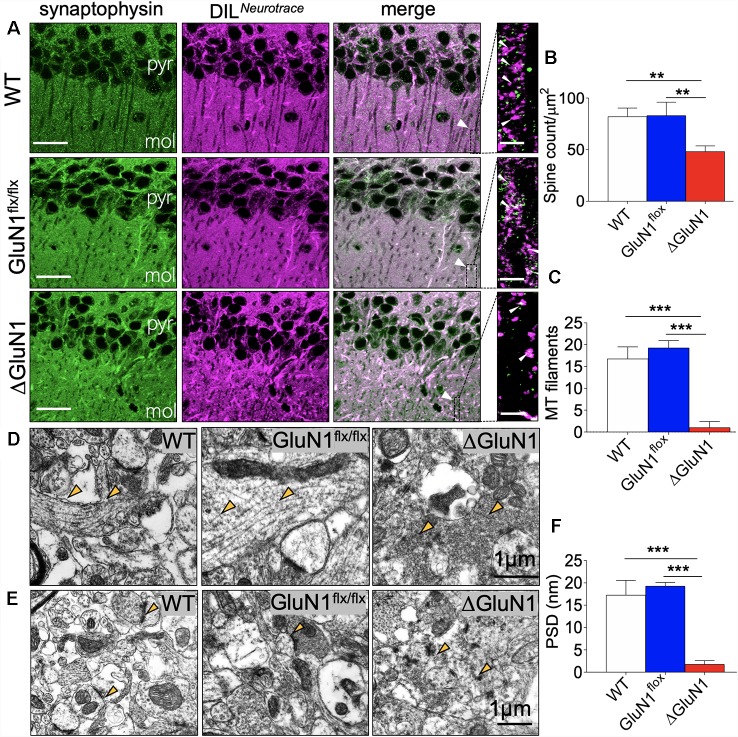
ΔGluN1 induced loss of dendritic spines in CA1 neurons. **(A)** Representative confocal images demonstrating co-localization of synaptophysin and DIL neurotracer in the CA1 (pyr, pyramidal layer; mol, molecular layer). Scale bar = 20 μm and 5 μm. **(B)** Bar graph [One-Way analysis of variance (ANOVA)] illustrating statistical comparison of CA1 dendritic spine count (DIL/synaptophysin). **(C)** Bar graph (One-Way ANOVA) comparing cytoskeletal filament count in hippocampal dendritic spines. **(D)** Transmission electron microscopy (TEM) photomicrographs illustrating hippocampal synapses. Yellow arrowheads indicate the synaptic cytoskeleton. Scale bar = 1 μm. **(E)** TEM photomicrographs demonstrating post-synaptic densities (PSD) in the hippocampus. Scale bar = 1 μm. **(F)** Bar graph illustrating the comparative thickness of PSDs (**B,C,F**; **p < 0.01, ***p < 0.001).

The general outline of dendritic spines was demonstrated using a combination of DIL Neurotracer, and synaptophysin immunofluorescence labeling (Figures 2A,B). Low magnification images demonstrate a significant increase in the distribution of DIL positive cytoskeletal aggregates in the CA1 of the ΔGluN1 hippocampus when compared with the WT and GluN1^flx/flx^. At higher magnification, this translates into a significant loss of dendritic spines in the ΔGluN1 hippocampus (Figure 2B; *p* < 0.01). To verify this outcome, dendritic spine morphology was further examined by ultrastructural imaging (TEM) of hippocampal synapses. As illustrated in Figures 2C,D, transmission electron photomicrographs revealed a prominent loss of cytoskeletal filament assembly (yellow arrowheads) in ΔGluN1 hippocampal synapse when compared with WT and GluN1^flx/flx^. In addition to the loss of dendritic spines and synaptic cytoskeletal perturbation, TEM images of the ΔGluN1 hippocampus also revealed a prominent decrease in the thickness of post-synaptic densities (Figures 2E,F; *p* < 0.001).

### Morphology of Dendrites

**Figure 3:**
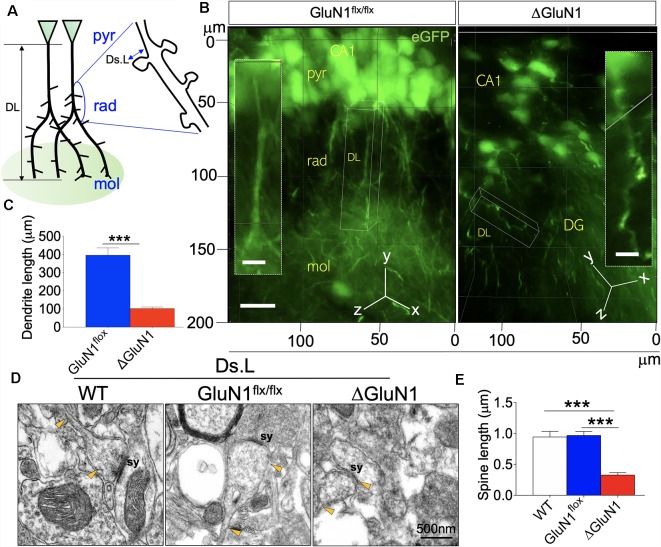
Length of dendrites and dendritic spine in the ΔGluN1 hippocampus.** (A)** Schematic illustration of dendrite length (DL) and dendritic spine length (Ds.L) for CA1 neurons (pyr, pyramidal layer; rad, radiatum layer; mol, molecular layer). **(B)** 3D-fluorescence images (eGFP) showing the outline of CA1 dendrites (pyr, pyramidal layer; rad, radiatum layer; mol, molecular layer). Scale bar = 20 μm, (inset) 5 μm. **(C)** Bar graph illustrating statistical comparison (*T*-test) of dendrite length. **(D)** TEM photomicrographs demonstrating dendritic spine morphology. Scale bar = 0.5 μm. **(E)** Bar graph (One-Way ANOVA) showing statistical comparison of dendritic spine length (TEM; **C,E**; ***p < 0.001).

The expression of membrane-bound eGFP conveyed by the AAV vectors was used to assess the morphology of the dendrites. Given that the expression of eGFP in the neuropil outlines dendrite branching, we used 3D-fluorescence imaging to demonstrate and measure the length of ΔGluN1 CA1 dendrites (μm). However, in order to assess the length of dendritic spines, we used ultrastructural synapse images acquired in TEM. Figure 3A illustrates the general outline of CA1 pyramidal neurons where DL and Ds.L represent the dendrite length and dendritic spine length, respectively. ΔGluN1 CA1 pyramidal neurons exhibits a significant decrease in dendrite length when compared with the GluN1^flx/flx^ (*p* < 0.001; Figures 3B,C). In assessing dendritic spine length (TEM), the distance between the dendrite shaft and the tip of the spine was determined (Figure 3A; Ds.L). The synapse (sy) in Figure 3D illustrates the tip of the dendritic spine where postsynaptic densities are present. Yellow arrowheads connote the dendritic spine shaft and synaptic expansion. In addition to a decrease in dendrite length (DL), the ΔGluN1 hippocampal neurons were also characterized by a decrease in dendritic spine length (Ds.L: *p* < 0.001; Figures 3D,E).

### Burst Activity in the CA1 Neural Network

**Figure 4:**
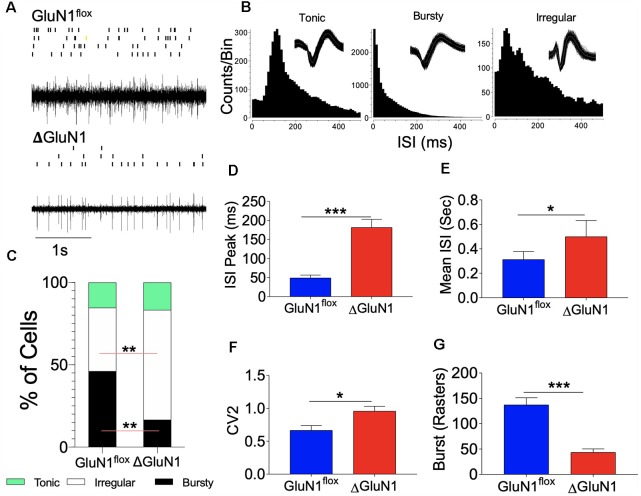
Interspike interval (ISI) characterization of CA1 spike train.** (A)** Single unit spike train and rasters recorded form the CA1 dendritic field. **(B)** Representative Interspike ISI histograms and waveform for tonic, bursty and irregular firing CA1 trains neuron units. **(C)** Composite bar graph depicting the percentage of CA1 neuron units based on ISI characterization of firing pattern.** (D–F)** Bar graphs illustrating ISI peak duration (ms), Mean ISI (ms), and CV_2_ of ISI for CA1 spike trains.** (G)** Bar graph illustrating the number of bursts recorded in the spike train per unit time (**D–G**; *p < 0.05, **p < 0.01, ***p < 0.001).

The relationship between the observed synaptic perturbations and neural encoding in the ΔGluN1 hippocampus was assessed by *in vivo* extracellular neural recording. Spontaneously evoked neural spikes in the CA1 dendritic field was analyzed to determine the impact of GluN1 loss of function on multi-synaptic burst encoding *in vivo*. Continuously recorded spike trains for *n* = 4 mice per group (Figure 4A) were sorted offline to isolate single units (GluN1^flx/flx^: *n* = 77 and ΔGluN1: *n* = 54) across six sessions. Spike train properties were assessed using the interspike interval (ISI) parameters which represent neural refractoriness over a time window. The firing property of each cell was determined with the ISI histogram (ISIH). Based on the ISIH properties, we grouped the neuron units as bursty, tonic, or irregular firing (Figure 4B). For the GluN1^flx/flx^ hippocampus, about 48% of the neurons exhibited distinct burst firing patterns while 38% are irregular firing neurons (*p* < 0.01). Conversely, in the ΔGluN1 hippocampus, 20% of the neurons showed characteristic burst firing pattern while ~60% were irregular firing neurons (*p* < 0.01; Figures 4B,C). Consistent with the suppression of burst firing, ΔGluN1 CA1 neurons exhibit a prolonged ISI peak duration (*p* < 0.001; Figure 4D), and an increased mean ISI duration (*p* < 0.05; Figure 4E).

In addition to a decreased burst firing, structural changes in ΔGluN1 hippocampal synapses was accompanied by an increased firing irregularity. Here, we determined the regularity of firing as the coefficient of variation (CV_2_) of the ISI in a fixed time window. A score of *CV*_2_ < 1 or *CV*_2_ = 1 depicts a regular firing pattern, while *CV*_2_ > 1 represents irregular firing between two fixed time points (τ_n_ to τ_n+1_). Regularity of firing based on the CV_2_ illustrates a time window computation of the ISI and is not affected by slight changes in firing frequency (spikes/s). Consistent with the percentage of irregular firing neurons, an average score of *CV*_2_ = 1.2 was recorded for the ΔGluN1 neurons when compared with the GluN1^flx/flx^ which showed significant firing regularity (*CV*_2_ = 0.5; Figure 4F). A prolonged ISI and increased irregularity of firing in the ΔGluN1 CA1 spike train was also associated with a decrease in burst count per unit time when compared with the GluN1^flx/flx^ spike train (*p* < 0.001; Figure 4G).

(1) $$CV_{2}=\frac{2[ISIn-ISIn+1]}{ISIn+ISIn+1}$$

### ΔGluN1-Linked Synaptic Perturbations Led to a Reduced Neuronal Firing Rate

**Figure 5:**
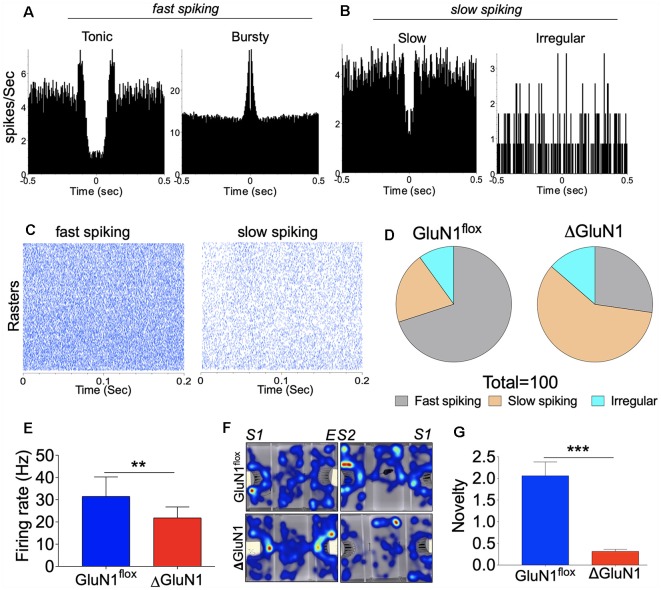
Firing rate (spikes/sec) characterization of the CA1 spike train.** (A,B)** Representative autocorrelogram for typical fast and slow spiking CA1 neuron units.** (C)** Perievent raster representation of fast and slow spiking CA1 units.** (D)** Pie chart depicting the percentage of fast, slow and irregularly firing CA1 neuron units. **(E)** Bar graph showing statistical comparison of mean firing rates (Hz).** (F)** Heat map demonstrating sociability and social novelty exploration pattern.** (G)** Bar graph illustrating social novelty memory index (**E,G**; **p < 0.01, ***p < 0.001).

In addition to ISIH characterization of spike train properties (bursty, tonic and irregular), we further examined the neuron population dynamics based on the percentage of *fast* or *slow* spiking neurons determined by autocorrelogram plots (Figures 5A,B). This was further verified by the peri-event raster plots which demonstrates number of events (ticks) per trial (Figure 5C) when the activity of all neurons (per session) was compared with a neuron with the highest raster count. By combining the autocorrelogram and peri-event raster plots, the neurons were further grouped based on their firing rate *r(t)* over a fixed time window (τ ms).

(2) r(t)=n/t

Together with ISIH characterization of neuron firing pattern, it was noted that the fast-spiking neurons were also bursty in their firing properties. On the other hand, irregular firing neurons exhibited a skewed autocorrelogram plot that connotes slow or desynchronized firing. As illustrated by the pie charts, the ΔGluN1 spike train exhibited a significant increase in the percentage of slow and irregularly firing neurons when compared with the GluN1^flx/flx^ spike train (*p* < 0.001; Figure 5D). Consequently, the mean firing rate *r(t)* for the ΔGluN1 CA1 network reduced significantly vs. the GluN1^flx/flx^ (*p* < 0.01; Figure 5E). In order to ascertain the impact of the highlighted synaptic changes on neural function, ΔGluN1 mice were assessed for sociability and social novelty. Heat maps in Figure 5F illustrates a significant decrease in sociability and social novelty performance index for ΔGluN1 mice when compared with the GluN1^flx/flx^. Together with the observed synaptic perturbations the ΔGluN1 mice also exhibited a decline in cognitive function (*p* < 0.001; Figure 5G).

### Cellular Basis of Firing Rate and ISI Dynamics in ΔGluN1 CA1

**Figure 6:**
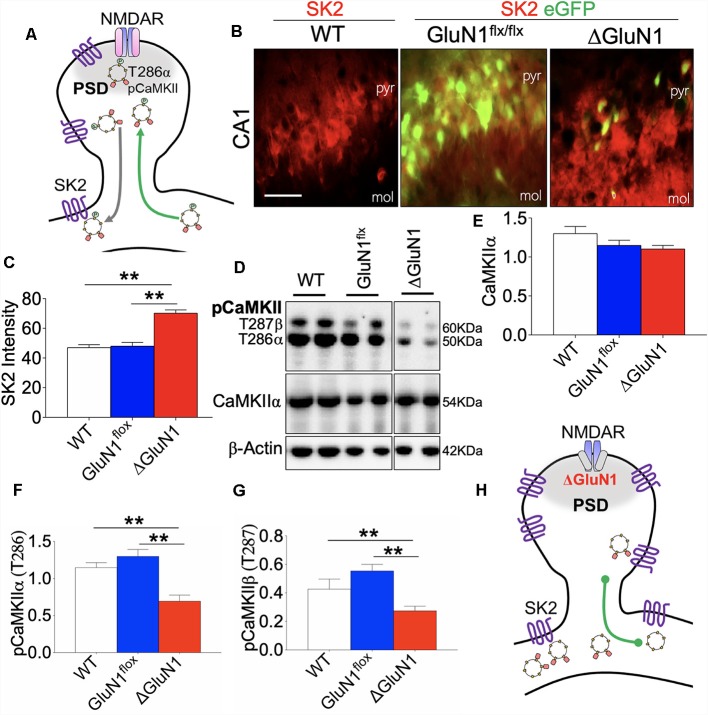
Co-dysregulation of SK2 and CaMKII in the ΔGluN1 hippocampus. **(A)** Schematic illustration of N-Methyl-D-Aspartate Receptor 1 (NMDAR), T286 pCaMKIIα, and SK2 co-localization at postsynaptic densities. **(B,C)** Representative fluorescence images and bar graph (One-Way ANOVA) demonstrating SK2 expression in the CA1 (pyr, pyramidal layer; mol, molecular layer). Scale bar = 20 μm **(B)**. **(D)** Representative immunoblots showing the expression of CaMKIIα, T286 pCaMKIIα and T287 pCaMKIIβ in whole hippocampal lysate. **(E–G)** Bar graphs showing statistical comparison of CaMKIIα (normalized with β-Actin), T286 pCaMKIIα, and T287 pCaMKIIβ (normalized with CaMKII). **(H)** Schematic representation of the ΔGluN1 hippocampal dendritic spine. T286 pCaMKIIα reduced significantly while SK2 expression is upregulated (**C,E,F,G**; **p < 0.01).

Synaptic potentiation in the hippocampus is mediated by several factors. However, it has been established that T286 phosphorylation of CaMKIIα and activation of SK2 can respectively enhance and attenuate synaptic activity (Figure 6A). Here, we showed that an increased ISI duration for the ΔGluN1 spike train was linked with a significant upregulation of hippocampal SK2 expression when compared with the controls (WT and GluN1^flx/flx^). Double labeling immunofluorescence in (Figures 6B,C) demonstrates a significant increase in SK2 expression for eGFP positive neurons in the ΔGluN1 CA1 (*p* < 0.01). Since SK2 activity determines the ISI, it is logical to speculate that the increased peak and mean of ISI duration (Figures 4D,E) were—in part—as a result of upregulated CA1 SK2 expression.

T286 pCaMKIIα synaptic localization and substrate targeting is required for LTP expression that is pertinent to long-term synaptic plasticity. As such, we determined the firing *r(t)* and burst discharge rate as indirect measures of synaptic T286 pCaMKIIα translocation efficiency in the CA1 neural network *in vivo*. In the ΔGluN1 hippocampus, the expression of CaMKIIα did not change significantly when compared with the control level (WT and GluN1^flx/flx^; Figures 6D–F). However, reduced firing (Figure 5E) and burst rates (Figure 4G) in the ΔGluN1 CA1 dendritic network was accompanied by a prominent decrease in T286 pCaMKIIα and T287 pCaMKIIβ (*p* < 0.01; Figures 6D–G; also Supplementary Figure S3). Together, ΔGluN1-induced dendritic spine perturbations and loss of neural plasticity involved the loss CaMKII activity and increased SK2 expression (Figure 6H).

### Activity-Coupled Regulation of SK2 and GluN1 Expression

**Figure 7:**
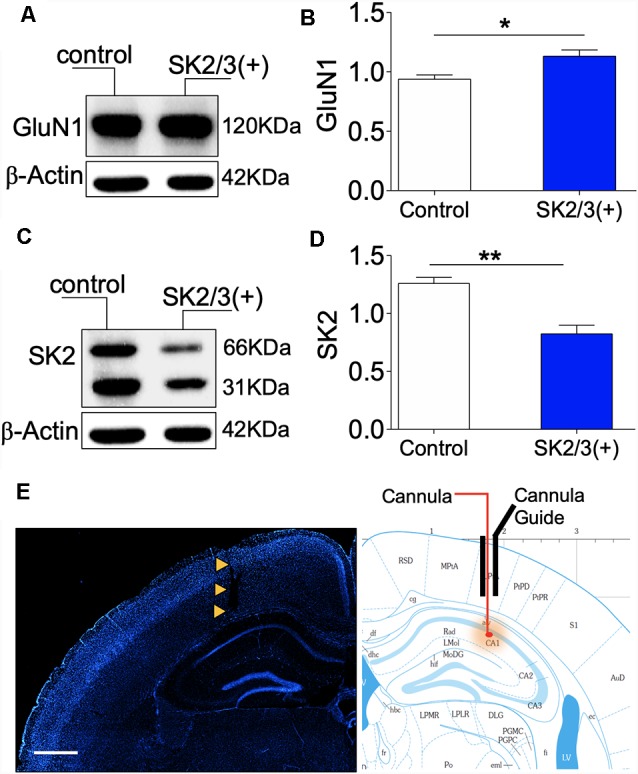
Activity-dependent expression of SK2 and GluN1. **(A,B)** Western blot and bar graph demonstrating hippocampal NMDAR-GluN1 expression. **(C,D)** Representative immunoblot and bar graph illustrating hippocampal SK2 expression. **(E)** Fluorescence image (DAPI) showing the track of the cannula guide and cannula for intrahippocampal injections. Scale bar = 0.5 mm (**E**; **B,D**; *p < 0.05, **p < 0.01).

While assessing synaptic function in the ΔGluN1 hippocampus, a notable change in spike train properties was an increase in the mean ISI duration (Figure 4E), and a reduced firing rate (Figure 5E). A possibility is that ΔGluN1 caused a decrease in transient Ca^++^ current that is necessary for SK2 activation and may cause SK2 overexpression through an activity coupled feedback mechanism. To test this hypothesis, we pharmacologically activated SK2 and determined the expression of GluN1 and SK2 by western blotting. Previously, we demonstrated that ablation of GluN1 (upstream) led to an increase in SK2 expression (Figure 6B). Here, we noted that positive modulation of SK2 caused a moderate increase in GluN1 expression (*p* < 0.05; Figures 7A,B), and significantly downregulate hippocampal SK2 expression (*p* < 0.01; Figures 7C,D; Supplementary Figure S4). These outcomes suggest that the expression of GluN1 and SK2 is inversely linked to their activity. Consequently, in the ΔGluN1 hippocampus, reduced Ca^++^ current required for the activation of SK2 may drive an increase in SK2 expression to compensate for the initial reduction in its activation. Likewise, repetitive activation of SK2-downstream of NMDAR—attenuates synaptic potentiation, and lead to an increase in GluN1 expression through a similar feedback mechanism. Figure 7E illustrates the site for stereotaxic implant of the cannula guide and drug delivery through the cannula.

### SK2-NMDAR Cross-Talk in the Regulation of CaMKIIα Activity

**Figure 8:**
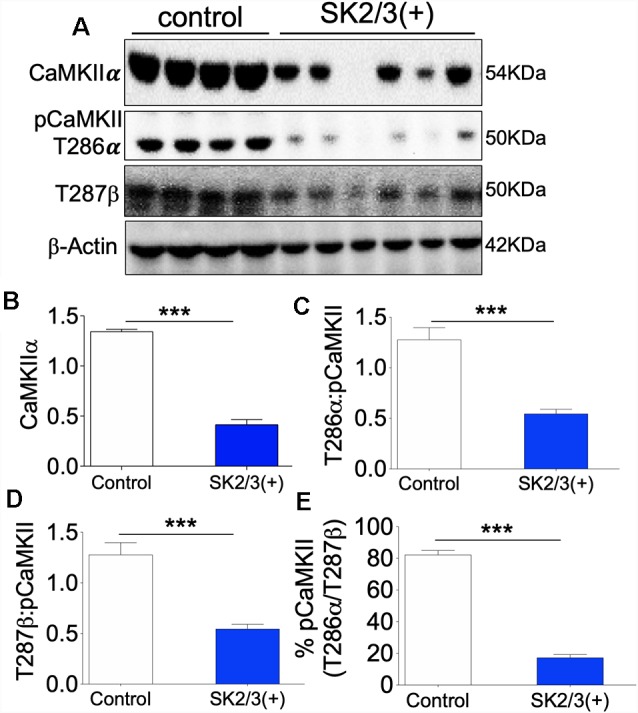
SK channel potentiation reduced hippocampal CaMKII expression (48 h after treatment *in vivo*). **(A)** Western blots demonstrating detected level of CaMKII, T286 pCaMKIIα, and T287 pCaMKIIβ in whole hippocampal lysate. **(B)** Bar graph representing comparative β-actin-normalized CaMKII expression (*T*-test). **(C,D)** Bar graph representing comparative CaMKII-normalized T286 pCaMKIIα and T287 pCaMKIIβ expression (*T*-test). **(E)** Bar graph demonstrating percentage T286/T287 (total) pCaMKII in the hippocampus (**B,C,D,E**; ***p < 0.001).

Given that the expression and activity of GluN1 and SK2 are tightly linked, an important question yet to be addressed is whether downstream positive modulation of SK channel can impact CaMKII activity and if such interaction might be dependent on NMDAR. In the ΔGluN1 hippocampus, T286/T287 phosphorylation of CaMKII decreased significantly. Although GluN1 expression was upregulated after a positive modulation of SK channel, the expression and T286 phosphorylation of CaMKIIα was significantly suppressed in the SK2/3(+) hippocampus (Figures 8A–C; *p* < 0.001; Supplementary Figure S5). Likewise, SK channel potentiation caused a significant decrease in T287 pCaMKIIβ (Figure 8D; *p* < 0.001). Ultimately, SK channel potentiation led to a decrease in percentage T286/T287 phosphorylated CaMKII in the hippocampus (Figure 8E; *p* < 0.001). These results are indicative of the role of SK2 in the tuning synaptic plasticity by CaMKII modulation.

### Positive SK Channel Modulation Impairs Synaptic Localization of CaMKII

**Figure 9:**
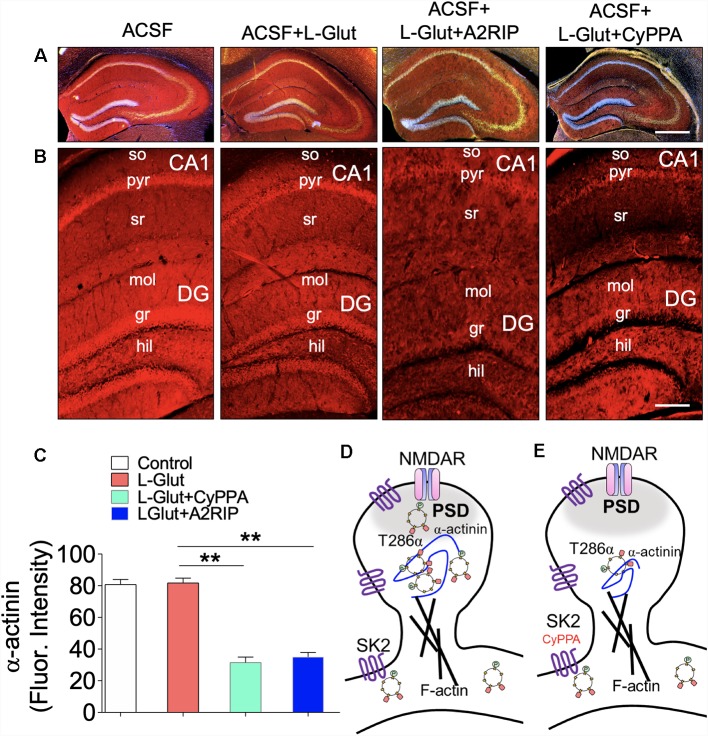
SK channel potentiation impacts hippocampal α-actinin expression. **(A–B)** Fluorescence images illustrating α-actinin expression in the hippocampus (so, stratum oriens; pyr, pyramidal layer; sr, stratum radiatum; mol, molecular layer; gr, granule cell layer, and hil, hilus). Scale bar = 0.4 mm **(A)**, 100 μm **(B)**. **(C)** Bar graph representation of α-actinin fluorescence intensity in the hippocampus (One-Way ANOVA). Schematic illustration of SK2 modulation of CaMKII spine anchorage: **(D)** synaptic pCaMKII is structurally linked with to the GluN2B sub-unit of NMDAR and is anchored to the dendritic spine cytoskeleton (F-actin) by α-actinin. In NMDAR-mediated synaptic potentiation, synaptic accumulation of pCaMKIIα/β is facilitated by α-actinin. **(E)** SK channel potentiation induced a loss CaMKII and α-actinin expression (**C**; **p < 0.01).

CaMKIIβ anchors the CaMKII (9α/3β) to dendritic spine F-actin by binding to α-actinin. There is substantial evidence to suggest that α-actinin—a F-actin binding protein—is pertinent to CaMKII (9α/3β) localization on synaptic cytoskeleton (Shen et al., 1998; Robison et al., 2005; Gustin et al., 2011; Jalan-Sakrikar et al., 2012; Lisman et al., 2012; Bosch et al., 2014; Hell, 2014; Khan et al., 2016). Consequent of the interaction between the CaMKIIα/β and α-actinin, the anchored CaMKII (9α/3β) determines the shape of the F-actin assembly, and structural plasticity of dendritic spines (Shen et al., 1998; Jalan-Sakrikar et al., 2012; Bosch et al., 2014; Hell, 2014). In acute slice preparation, perfusion with Ca^++^-ACSF containing L-Glutamate caused no significant change in hippocampal α-actinin expression when compared with ACSF only (Figures 9A–C). However, when a CaMKII inhibitor (A2RIP) or SK channel positive modulator (CyPPA) is paired with L-Glutamate, there was a significant loss of α-actinin in the hippocampus (*p* < 0.01; Figure 9C). Based on these outcomes, we propose that SK2 may suppress long-term synaptic plasticity by attenuating synaptic localization of T286 pCaMKIIα (Figures 9D,E).

### Compartmental Localization of CaMKII in the Hippocampus

**Figure 10:**
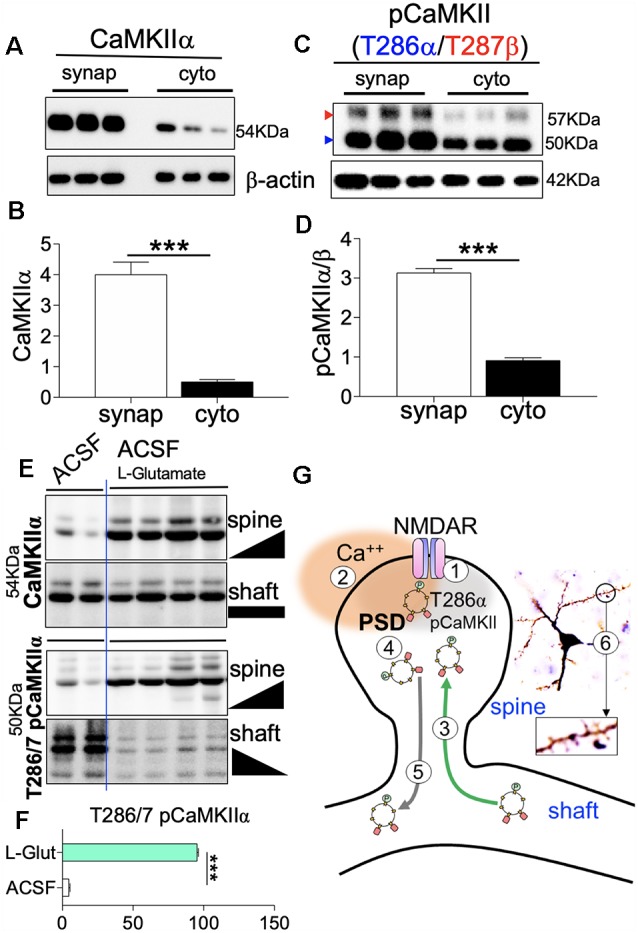
Synaptic potentiation increase spine (synaptic) translocation of CaMKII. **(A–D)** Representative western blots and bar graphs demonstrating the distribution of CaMKII and T286 pCaMKIIα/T287 pCaMKIIβ in synaptosomal and shaft-cytosol tissue extracts derived from WT hippocampus. **(E)** Representative immunoblots illustrating the comparative synaptic (spine) and shaft-cytosol expression of CaMKII and T286/T287 pCaMKIIα/β. **(F)** Bar graph (*T*-test) illustrating the synaptic translocation of T286/T287 pCaMKIIα/β post L-Glutamate treatment. **(G)** Schematic illustration of synaptic translocation of T286/T287 pCaMKIIα/β. (1) NMDAR potentiation; (2) post-synaptic Ca^++^ transient activates SK2; (3) Ca^++^ transient facilitates T286/T287 CaMKII phosphorylation, and synaptic translocation; (4) increased SK2 activity facilitates removal of synaptic T286/T287 pCaMKIIα/β; and (5) translocation of CaMKII from dendritic spine to shaft-cytosol. (**B,D,F**; ***p < 0.001).

We processed microdissected whole hippocampus to isolate synaptosomal extracts containing post-synaptic densities (PSD), and cytosolic extracts containing dendritic shaft. We validated the tissue extracts by western blotting to detect PSD proteins—PSD-95 and GluN1—in the synaptosomal extract. Likewise, the shaft-cytosol extract was validated by immunoblot detection of ErK1/2 and pErK1/2. The validation protocol was set up as a reciprocal detection experiment where synaptic proteins are also assessed in shaft-cytosol extracts, and vice versa. For all samples, PSD-95 (S2.1,S2.2) and GluN1 (S2.3,S2.4) were enriched in the synaptosomal extract, and not in the shaft-cytosol domain. Similarly, ErK1/2 (S2.5,S2.6) and pErK1/2 (S2.7,S2.8) were enriched in the shaft-cytosol domain only. In order to determine compartmental localization of CaMKIIα, we performed immunoblot detection of CaMKIIα, and T286α/T287β phosphorylated isoforms in the synaptosome and shaft-cytosol extracts. Our results revealed that CaMKIIα is predominantly localized in the synaptic compartment when compared with the shaft-cytosol (Figures 10A,B; *p* < 0.001). Likewise, T286 pCaMKIIα and T287 pCaMKIIβ were also enriched in the synaptic compartment when compared with the shaft-cytosol extract (Figures 10C,D; *p* < 0.001).

In subsequent experiments, we incubated the microdissected hippocampus in glucose-rich oxygenated Ca^++^-ACSF to determine how an *ex vivo* treatment might affect the compartmental localization of CaMKII (Figures 10A–D). After 1 h of incubation in ACSF, CaMKIIα and T286α/T287β were predominantly localized in the shaft-cytosol domain (Figure 10E). However, when 10 μM L-Glutamate is included the ACSF, CaMKIIα was predominantly localized in the synaptic compartment, and was accompanied by a near total T286α/T287β synaptic translocation (Figures 10E,F; *p* < 0.001). These results suggest that L-Glutamate-driven synaptic potentiation is required for spine recruitment of CaMKIIα and T286α/T287β (Figure 10G).

### SK Channel Potentiation Reduced the Efficiency of CaMKII Translocation

**Figure 11:**
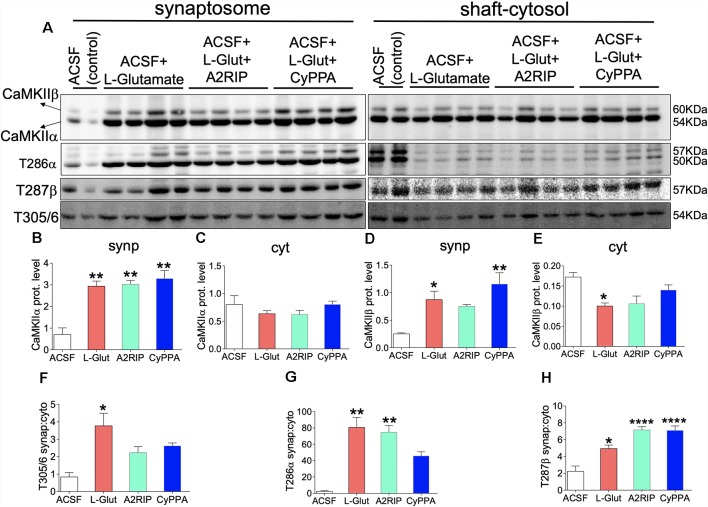
SK channel potentiation impacts hippocampal synaptic translocation of T286/T287 pCaMKIIα/β. **(A)** Representative western blots illustrating synaptosomal and shaft-cytosol level of CaMKIIα, CaMKIIβ, T286 pCaMKIIα, T287 pCaMKIIβ, and T305/306 pCaMKII. Bar graphs illustrating; **(B)** synaptosomal expression of CaMKIIα. **(C)** Shaft-cytosol expressions of CaMKIIα. **(D)** Synaptosomal expression of CaMKIIβ. **(E)** Shaft-cytosol expressions of CaMKIIβ. **(F)** Level of normalized synaptic T305/306 pCaMKII relative to shaft-cytosol expression. **(G)** Level of normalized synaptic T286 pCaMKIIα relative to shaft-cytosol expression. **(H)** Level of normalized synaptic T287 pCaMKIIβ relative to shaft-cytosol expression (**B–H**; *p < 0.05, **p < 0.01, ****p < 0.0001).

Figure 8 demonstrates a significant loss of neural CaMKII, T286α and T287β after SK channel potentiation *in vivo* (48 h). In order to determine the short-term (1 h) effect of SK channel on synaptic localization of CaMKII, we paired L-Glutamate treatment with SK channel potentiation in acute preparations (Figure 11A). L-Glutamate treatment significantly increased synaptic localization of CaMKIIα (*p* = 0.0031; Figure 11B). Normalized synaptic expression of CaMKIIα also increased when L-Glutamate was paired with CaMKII inhibitor (A2RIP; *p* = 0.0024) or SK channel potentiator (*p* = 0.0010; Figure 11B). Similar to L-Glutamate treatment, a combination of L-Glutamate with A2RIP or CyPPA did not impact the shaft-cytosol localization of CaMKIIα (Figure 11C). L-Glutamate treatment increased synaptic localization of CaMKIIβ when compared with ACSF only (*p* = 0.0216; Figure 11D). However, when combined with CyPPA, the synaptic translocation of CaMKIIβ was significantly upregulated (*p* = 0.0015). L-Glutamate treatment caused a significant decrease in shaft-cytosol localization of CaMKIIβ when compared with ACSF (*p* = 0.0453). This was attenuated by SK channel potentiation and CaMKII inhibition. For these treatment combinations, there was no significance vs. ACSF (baseline; A2RIP and CyPPA; Figure 11E).

In order to ascertain the effect of SK channel potentiation (CyPPA) on CaMKII translocation efficiency, we normalized synaptosomal pCaMKII (T286, T287, T305/306) with shaft-cytosolic expression level. While L-Glutamate significantly increased synaptosomal translocation of T305/306 pCaMKII (*p* = 0.0158), pairing L-Glutamate with either CaMKII inhibition or SK channel potentiation suppressed synaptic translocation of T305/306 (Figure 11F). Accordingly, when L-Glutamate is paired with A2RIP or CyPPA, there is no significant change in synaptic T305/306 expression vs. ACSF (Figure 11F). L-Glutamate treatment increased the synaptic localization of T286 pCaMKIIα when compared with ACSF (*p* = 0.0016). Similar to T305/306, pairing L-Glutamate with SK channel potentiation reduced synaptic T286 pCaMKIIα localization (Figure 11G). Here, the significance recorded for L-Glutamate—vs. ACSF only—was abolished when L-Glutamate is paired with CyPPA (ns). L-Glutamate treatment also increased the synaptic localization of T287 pCaMKIIβ when compared with ACSF only (*p* = 0.0170; Figure 11H). As shown in Figure 11D, CyPPA treatment induced a more prominent increase in synaptic CaMKIIβ expression (*p* = 0.0015) when compared with L-Glutamate (*p* = 0.0216); vs. ACSF. To this effect, pairing L-Glutamate with CyPPA also caused a prominent increase in synaptic T287 pCaMKIIβ (*p* < 0.0001) when compared with L-Glutamate (*p* = 0.0170); vs. ACSF (Figure 11H). Together, our results suggest that SK channel potentiation suppressed synaptic translocation of T286 pCaMKIIα while increasing T287β pCaMKII phosphorylation in acute experiments.

### SK Channel Potentiation Modulates CA1 Burst Encoding

**Figure 12:**
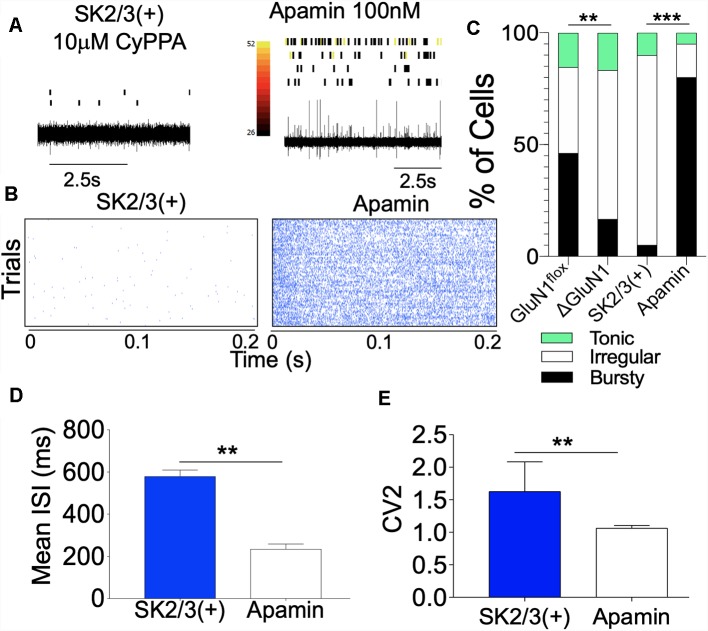
SK channel potentiation attenuates spontaneous CA1 firing. **(A)** Spike train with aligned rasters demonstrating CA1 dendritic field neural activity. **(B)** Perievent rasters demonstrating a change in neural activity following SK channel potentiation and inhibition. **(C)** Composite bar graph demonstrating percentage distribution of neuron units characterized by ISI properties. **(D)** Bar graph demonstrating Mean ISI (ms) duration. **(E)** Bar graph demonstrating regularity of firing (CV_2_ of ISI). (**C–E**; **p < 0.01, ***p < 0.001).

The physiological implication of a suppressed T286/T287 phosphorylation and imbalanced translocation to the synapse is that LTP may be abrogated. In addition, long-term synaptic plasticity modulated by CaMKII may become significantly dysregulated. In a separate experiment, we examined the effect of SK channel positive modulation (10 μM CyPPA) or inhibition (100 nM Apamin) on CA1 dendritic neural network burst encoding *in vivo*. In anesthetized mice, SK channel agonist (CyPPA) was infused, and spontaneously evoked spikes were recorded with a tetrode. Subsequently, Apamin (SK2 blocker) was infused to attenuate the activity of SK2 in the CA1 dendritic field. As illustrated in Figure 12A, positive modulation of SK channel function significantly reduced spontaneous CA1 firing while Apamin treatment rescued CA1 firing and burst activity (Figures 12A,B). Based on ISI characterization of firing pattern (Figures 4B,C), there was a significant increase in the percentage of irregular firing neurons and a decrease in bursty neurons for SK2/3(+) CA1 spike train (*p* < 0.001; Figure 12C). Subsequent Apamin treatment increased the percentage of cells with characteristic bursty firing pattern, while also reducing the percentage of irregular firing neurons (Figure 12C; *p* < 0.001). Further evaluation of the ISI revealed that SK2 blockade by Apamin reduced the mean ISI duration (*p* < 0.01; Figure 12D) and increased the regularity of firing (Figure 12E) of CA1 neurons. This was seen as a decrease in CV_2_ score for the ISI (*p* < 0.01) following Apamin infusion.

### SK Channel Modulation of Firing Rate

**Figure 13:**
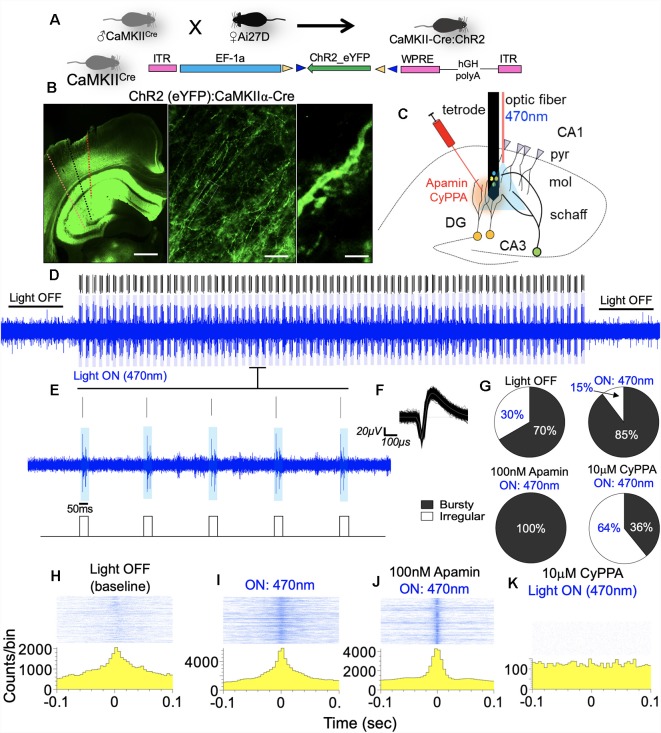
SK channel modulation of CA1 burst discharge. **(A)** Schematic illustration of a double floxed AAV-ChR2-eYFP expression in CaMKIIα-Cre hippocampus. **(B)** Reporter eYFP—fluorescence in the hippocampus of CaMKII-Cre:ChR2 mouse. **(C)** Schematic illustration of an experimental set up depicting the relative position of the optic fiber, drug injection, and recording electrode shank (pyr, pyramidal layer; mol, molecular layer; schaff, schaffer collateral). Scale bar = 0.5 mm, 50 μm, 5 μm. **(D,E)** Spike train with adjoining rasters demonstrating 470 nm LED light pulse (50 ms) at 1 Hz. **(F)** Sample waveform for spikes generated by optogenetic stimulation of CA1 neuron units. **(G)** Pie chart representing ISI histogram characterization of neurons based on firing properties. **(H–K)** Sample perievent histogram demonstrating rhythmicity of neuronal population during photostimulation, and when paired with SK channel modulation *in vivo*.

Here, we measured CA1 network firing rate (Hz) when pyramidal cell dendritic field photostimulation is paired with pharmacological modulation of SK2 *in vivo*. Figures 13A,B illustrates the expression of ChR2 in the hippocampus and dendritic spine. Dotted lines represent the electrode/optic fiber track in the hippocampus (Figure 13B). The experimental set up for placement of the recording electrode, optic fiber, and needle for drug infusion is schematically illustrated in Figure 13C. Blue light pulse (470 nm) was delivered by a square wave (TTL) generator at 1 Hz (50 ms) to potentiate the spines. The effect of the photostimulation regime is demonstrated by the 470 nm light ON phase (Figures 13D–F). When the ISI histogram was used for the characterization of the firing pattern, ChR2 photostimulation increased burst firing as indicated by the percentage of bursty neurons (Figure 13G; *p* < 0.001). Interestingly, when SK2 inhibition (Apamin) is paired with photostimulation, the threshold of bursting activity increased drastically with ~100% of neurons showing burst activity. However, when photostimulation is paired with SK channel potentiation, a significant decrease in burst activity was recorded (*p* < 0.001). This is further evident in perievent histogram plots that depict response strength and synchrony of CA1 synaptic units. A paired photostimulation and SK2 inhibition regime increased the firing rate and synchrony when compared with photostimulation only. Conversely, SK channel activation by CyPPA scrambled the synchrony and response strength of the CA1 units (Figures 13H–K).

**Figure 14:**
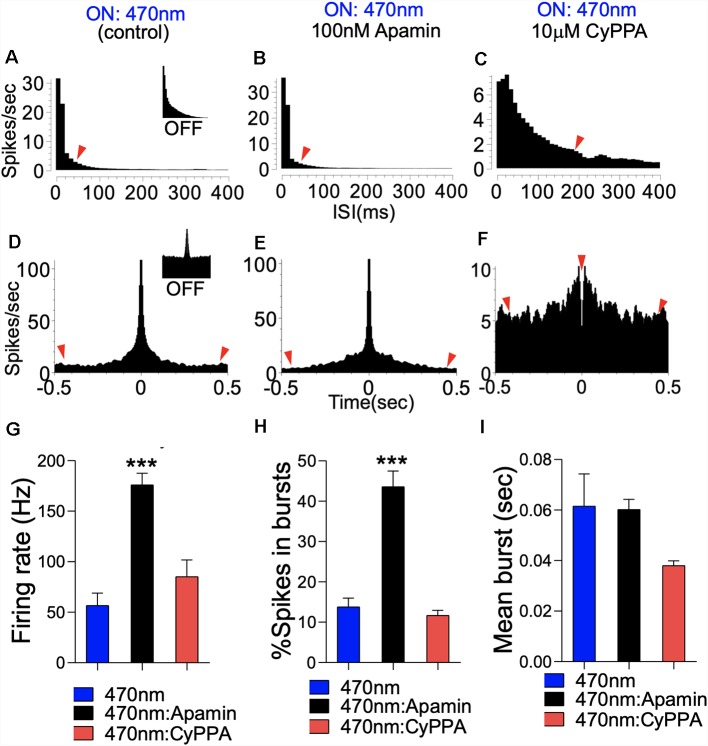
SK modulation of burst firing in CA1 dendritic neural network. **(A)** Representative ISI histogram for a bursty CA1 neuron unit following optogenetic stimulation. Inset (Light Off). **(B)** ISI histogram illustrating a prominent increase in burst firing when photostimulation is paired with SK2 inhibition (Apamin). **(C)** ISI histogram illustrating scrambled firing pattern when photostimulation is paired with SK channel potentiation (CyPPA). **(D–F)** Autocorrelogram demonstrating a monosynaptic/pyramidal firing pattern in the CA1 neural network, and the effect of SK channel modulation. Inset (**D**; Light OFF). **(G)** Bar graph illustrating a change in firing rate. **(H)** Bar graph illustrating a change in percentage spikes in a burst. **(I)** Bar graph demonstrating the mean burst duration (**G–I**; ***p < 0.001).

The significance of this outcome is further evident in the analysis of the ISI histogram plot. For paired photostimulation and SK channel inhibition, the ISI histogram shows strong burst activity that is characterized by a decreased ISI, and rapid onset for ISI decay (red arrow head; Figures 14A,B). On the other hand, pairing SK channel potentiation with photostimulation prolonged the ISI duration, and delayed the onset of ISI decay (>400 ms; Figure 14C). This outcome is further supported by analysis of autocorrelogram which revealed a prominent increase in firing strength as a result photostimulation (Figure 14D; red arrow heads); compared with spontaneously firing bursty neuron (inset). Pairing photostimulation and SK2 inhibition further enhanced burst strength of CA1 network as illustrated by a further depression of the edge of autocorrelogram plot (Figure 14E). However, when photostimulation is coupled with SK channel potentiation, synaptic response strength and synchrony decreased significantly as shown by a decrease in autocorrelogram peak strength (Figure 14F). This is consistent with an increased ISI duration (ms; Figure 14C), and reduced synchrony of firing shown by the perievent raster plot (Figure 13K). Suppression of SK2 activity by Apamin during photostimulation increased the firing rate and the number of spikes in a burst when compared with photostimulation only (Figures 14G,H, *p* < 0.0001). As expected, pairing SK channel potentiation with photostimulation abolished the firing rate increase recorded in photostimulation only (Figures 14G,H). Interestingly, our results revealed that the mean burst duration was not altered irrespective of the paired SK2 modulation event. Based on these outcomes, we deduced that the dendritic SK channel modulates burst encoding by directing the firing rate and number of spikes in a burst. While these parameters are linked to the probability of burst firing, we noted that SK channel modulation did not alter the duration of burst events in the CA1 neural network.

## Discussion

NMDAR is a glutamate type ionotropic receptor that is relatively abundant in the hippocampus and mediates LTP (Hatton and Paoletti, 2005; Coultrap and Bayer, 2012; Lisman et al., 2012; Babiec et al., 2017). A change in the expression of NMDAR or loss of function of its sub-units (GluN1 and GluN2A and 2B) have been identified in the etiology and progression of neuropsychiatric disorders (Hansen et al., 2014; Huang and Gibb, 2014; Wesseling et al., 2014; Bustos et al., 2017). Neurocognitive disorders such as autism, schizophrenia, and depression exhibit various forms of NMDAR hypofunction (Duffney et al., 2013; Snyder and Gao, 2013; Cohen et al., 2015; Zhou et al., 2016; Gulchina et al., 2017; Nakazawa et al., 2017; Ogundele and Lee, 2018; Rebollo et al., 2018). Although the causes of developmental neuropsychiatric defects are wide and varied, recent evidence suggests that loss of NMDAR function is a determinant of the synaptic and behavioral defects.

In LTP, synaptic potentiation by NMDAR-linked ionotropic neurotransmission is regulated through various mechanisms. Notably, positive modulation of synaptic strength which is pertinent to memory encoding is facilitated by CaMKIIα T286 phosphorylation (Gustin et al., 2011; Coultrap and Bayer, 2012; Lisman et al., 2012; Coultrap et al., 2014). On the other hand, negative modulation of synaptic strength could be driven by small conductance (SK2) ion channel (Ngo-Anh et al., 2005; Hammond et al., 2006; Maingret et al., 2008). The NMDAR-mediated Ca^++^ current constitutes 75% of post-synaptic transient that is central to synaptic potentiation and LTP. The mechanism involves activation (T286 autophosphorylation) of a closed CaMKII structure (T305/T306) to promotes kinase activity and substrate targeting (Lisman et al., 2002, 2012). In the T286 autophosphorylated state, CaMKII holds an increased (10-folds) affinity for Ca^++^-calmodulin binding, and promotes high-frequency firing that leads to LTP expression (Gustin et al., 2011; Coultrap and Bayer, 2012; Hell, 2014; Ma et al., 2015). On the other hand, activation of SK2 by the Ca^++^ surge suppresses neuronal firing by spike frequency adaptation that is pertinent to intrinsic excitability regulation (Ngo-Anh et al., 2005; Hammond et al., 2006; Lin et al., 2008; Lee and MacKinnon, 2018). Mechanistically, the activity of SK2 constitutes the after-hyperpolarization phase, that depicts the interspike interval, during which a synaptic unit is least expected to fire (Stackman et al., 2002, 2008; Ngo-Anh, 2006; Kim and Hoffman, 2008; Lin et al., 2008; Maingret et al., 2008; Prescott and Sejnowski, 2008; Toporikova and Chacron, 2009; Kuiper et al., 2012; Trimmer, 2015). Based on these concepts, NMDAR hypofunction may also imply—to an extent—the suppression of CaMKIIα T286 phosphorylation and (or) a positive modulation of SK2 function. Consistent with this idea, genetic ablation of NMDAR or pharmacological potentiation of SK2 suppressed T286 phosphorylation of CaMKIIα and reduced the firing rate of CA1 neurons *in vivo*.

The frequency of NMDAR Ca^++^ transients represents a significant aspect of synaptic potentiation that impacts dendritic spine plasticity in the hippocampus. As such, the structure of dendritic spines, distribution of dendritic spines, and CA1 network firing pattern are directly related to LTP expression and structural plasticity (Jauregui et al., 2017; Nakahata and Yasuda, 2018). While an increased CaMKII T286 phosphorylation is characteristic of high-frequency neuronal firing, an increased SK2 function attenuates neuronal firing. Given that NMDAR activation produces the transient Ca^++^ current that activates CaMKII and SK2 for synaptic potentiation and depression respectively, a significant aspect of this interaction that is yet to be considered is whether SK2 refine synaptic plasticity by modulating the spine dynamics of CaMKII. Taken together, our results demonstrate that genetic ablation of GluN1 in the hippocampus significantly perturbed dendritic spine morphology, and attenuate CA1 burst firing *in vivo*. This is attributable—in part—to the activity-coupled upregulation of SK2 expression, and a suppression of T286 CaMKIIα phosphorylation. Interestingly, in the presence of normal GluN1 function, positive modulation of SK channel equally reduced hippocampal synaptic T286 pCaMKIIα localization.

### Cytoskeletal Anchorage of CaMKII

Our results demonstrate that SK channel potentiation *in vivo* caused prominent loss of CaMKII, and suppressed T286/287 phosphorylation after 48 h. However, in acute treatment (1 h) performed with *ex vivo* slices, we found that SK channel potentiation disrupts T286 pCaMKIIα and T287 pCaMKIIβ synaptic homeostasis. It follows that the CaMKII heteromeric dodecamer (9α/3β) is anchored to dendritic spine cytoskeleton by an F-actin binding protein—α-actinin (Shen et al., 1998; Robison et al., 2005; Jalan-Sakrikar et al., 2012; Khan et al., 2016). However, it has been previously established that CaMKIIβ holds a stronger affinity, and is primarily involved in anchoring the holoenzyme α-actinin/F-actin (Coultrap and Bayer, 2012). T287β phosphorylation allows the holoenzyme to detach from the cytoskeletal anchor and facilitates substrate targeting (kinase activity) of the T286 pCaMKIIα component. Here, our results demonstrate that SK channel potentiation suppresses synaptic localization of T286 pCaMKIIα while promoting T287β phosphorylation (Figures 11G,H). The physiological implication is that the synaptic substrate activity (T286 pCaMKIIα) and cytoskeletal anchorage (CaMKIIβ) are significantly reduced following SK channel potentiation. The latter is further evident by the prominent loss of α-actinin in the hippocampus after SK channel potentiation (Figure 9).

### SK2 in CA1 Burst Encoding

Spontaneously evoked synaptic potentials are necessary for hippocampal neural plasticity and are representative of the state of CA1 neural network (Winnubst et al., 2015). Here, neurons were characterized using the shape of the ISIH which is mostly dependent on SK2 function and represents a distinct firing signature based on repetitive patterns of refractoriness (Maylie et al., 2004; Ngo-Anh et al., 2005; Hammond et al., 2006). Additionally, we assessed burst and firing rate events that are dependent on the threshold of T286 pCaMKIIα synaptic localization (Coultrap and Bayer, 2012; Hell, 2014; Penny and Gold, 2018). Together with the structural perturbations of dendritic spine, our results suggest that loss of GluN1 function may also connote the suppression of CaMKII T286 phosphorylation and upregulation of SK2 function (Figure 6H). Here, we showed that the suppression of CaMKII function in the ΔGluN1 hippocampus was associated with a reduced burst firing (Figure 4). Likewise, upregulated SK2 expression may underlie an increase in the ISI duration within the CA1 neural network (Figures 4, 12).

Spontaneously evoked neural spikes recorded from the CA1 dendritic field were characterized by mean a firing rate of 30 spikes/s (Figure 5). As a result of GluN1 loss of function (ΔGluN1), there was a significant decrease in firing rate. This is further supported by an increased percentage of irregularly firing neurons, and a decrease in the count of bursty neuron units. To this effect, loss of GluN1 function in the hippocampus was associated with an increased irregularity of firing which was determined by the coefficient of variation (CV_2_) of the ISI. Determining the regularity of firing based on CV_2_ is solely dependent on the ISI and is not affected by a slight change in firing frequency. Since SK2 function impacts the ISI, the CV_2_ over a time window may be representative of the effect of SK2 on firing regularity. To support this outcome, in normal GluN1 function (Figure 12), potentiation of SK2 reduced the regularity of firing while suppressing the firing rate.

In addition to an increased ISI, positive modulation of SK2 also suppressed burst and firing rates in the CA1 neural network (Figures 12A,B). In a typical synaptic potentiation event, high-frequency firing is dependent on NMDAR Ca^++^ transient, and CaMKIIα T286 phosphorylation at postsynaptic densities. Here, we noted that SK channel potentiation reduced the fringing rate—number of spikes per unit time (n/t)—by suppressing synaptic localization of T286 pCaMKIIα (Figures 8, 11). Although GluN1 ablation led to a loss of T286 pCaMKIIα (Figures 6D,E), the associated upregulation of SK2 likely enhanced the suppression of T286 pCaMKIIα synaptic localization.

### SK2 Regulation of Synaptic Plasticity Involves CaMKII Modulation

Our results suggest that GluN1 regulation of synaptic plasticity is dependent on a balanced activity of SK2 and CaMKII in hippocampal dendritic spines. Activation (T286 phosphorylation) of CaMKIIα promotes synaptic potentiation, and is a determinant of neuronal firing rate (n/t), and burst activity (Giese et al., 1998; Coultrap and Bayer, 2012; Coultrap et al., 2014). A coupled activation of SK2 by Ca^++^ transient determines the pattern of neural refractoriness and duration between successive spikes (ISI). Since SK2 modulate the ISI duration, it is also directly involved in the regulation of burst rates (Stackman et al., 2002, 2008; Lin et al., 2008; Maingret et al., 2008). As a result, there is a possibility of an overlap in CaMKII activity and SK2 function in LTP. Consistent with the previously described changes in dendritic spine morphology, we noted that an increase in ISI inversely correlated with both regularity of firing (Figure 4F) and the firing rate in the ΔGluN1 CA1 network (Figure 5E). Likewise, when SK2 was activated in the WT hippocampus (normal GluN1 function), both the firing rate and regularity of firing reduced significantly (Figure 12); similar to ΔGluN1. In addition to a decreased firing rate, positive modulation of SK channel function led to an abrupt suppression of CaMKIIα and its T286 phosphorylation in the hippocampus (Figure 8). To this effect, a subsequent infusion of SK2 blocker—Apamin—increased neuronal firing rate and percentage bursty neurons when compared with the SK channel potentiation.

### SK2 Modulation of Firing Rate and Neural Burst Discharge

Synaptic translocation of T286 pCaMKIIα is dependent on the stimulation frequency. Accordingly, high-frequency stimulation events facilitate a rapid synaptic T286pCaMKIIα recruitment (LTP) compared to low-frequency stimulation (Håvik et al., 2003; Coultrap and Bayer, 2012; Coultrap et al., 2014; Chang J. Y. et al., 2017). Since positive modulation of SK channel activity attenuate hippocampal T286 pCaMKIIα localization (Figure 8) and burst discharge rate (Figure 12), we ask whether SK modulation of CA1 firing properties is dependent on the stimulation state. Photostimulation of genetically encoded ChR2 in the hippocampus of mice increased burst activity and synaptic strength when the ISI histogram and autocorrelogram plots were evaluated (Figures 13H, 14). Interestingly, Apamin-induced SK2 blockage further enhanced burst firing and the firing rate of CA1 neurons when compared with photostimulation only. In the absence of an external stimulus *in vivo*, spontaneously evoked CA1 firing rate and burst rate were significantly attenuated during SK channel potentiation (Figure 12). Likewise, when we paired photostimulation with SK channel potentiation, it significantly abolished the burst and firing rate increase associated with the photostimulation event (Figures 14G–I).

## Summary

Together, our results suggest that GluN1 modulation of spine plasticity and neuronal firing involves the co-regulation of SK2 and CaMKII. Additionally, SK2 can tune long-term synaptic plasticity by refining spine-specific localization and activity of CaMKII.

## Ethics Statement

All animal handling procedures were approved by the Institutional Animal Care and Use Committee (IACUC) of the Louisiana State University (LSU) School of Veterinary Medicine (SVM).

## Author Contributions

OO and CL designed the experiments and prepared the manuscript. OO, AS and RS conducted the experiments and analyzed the results. OO, CL, RS and AS checked the manuscript.

## Conflict of Interest

The authors declare that the research was conducted in the absence of any commercial or financial relationships that could be construed as a potential conflict of interest.
