# Supplementary material for: SK Channel Modulates Synaptic Plasticity by Tuning CaMKIIα/β Dynamics
Source: Front Synaptic Neurosci. 2019 Oct 31;11:18. doi: 10.3389/fnsyn.2019.00018 (PMC6834780; doi:10.3389/fnsyn.2019.00018)
Supplement: Supplementary file 1 [file Data_Sheet_1.docx]

**Supplemental information**


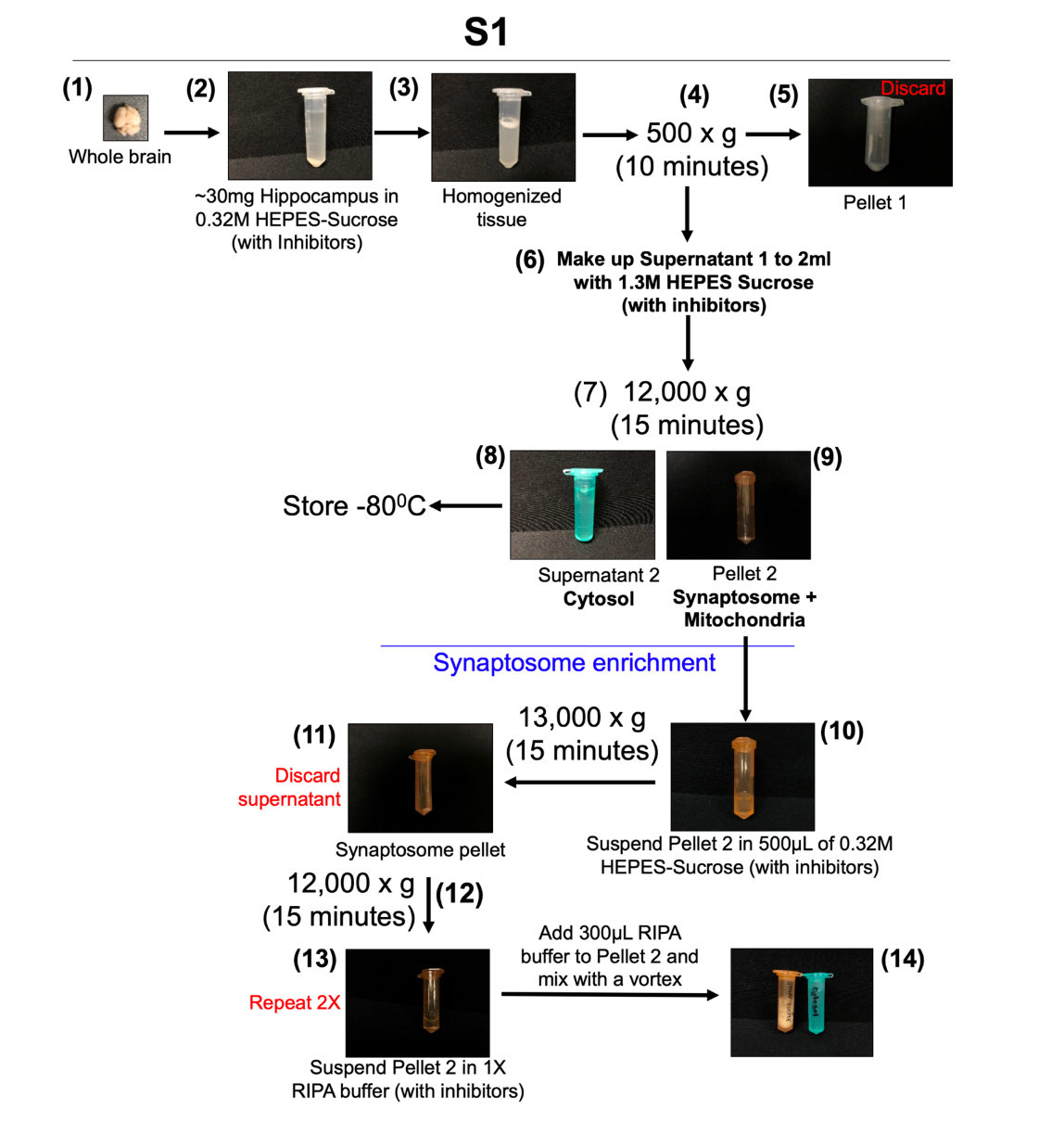


**Figure S1**

Schematic illustration of sucrose gradient method for synaptosome and cytosol isolation form hippocampal lysate


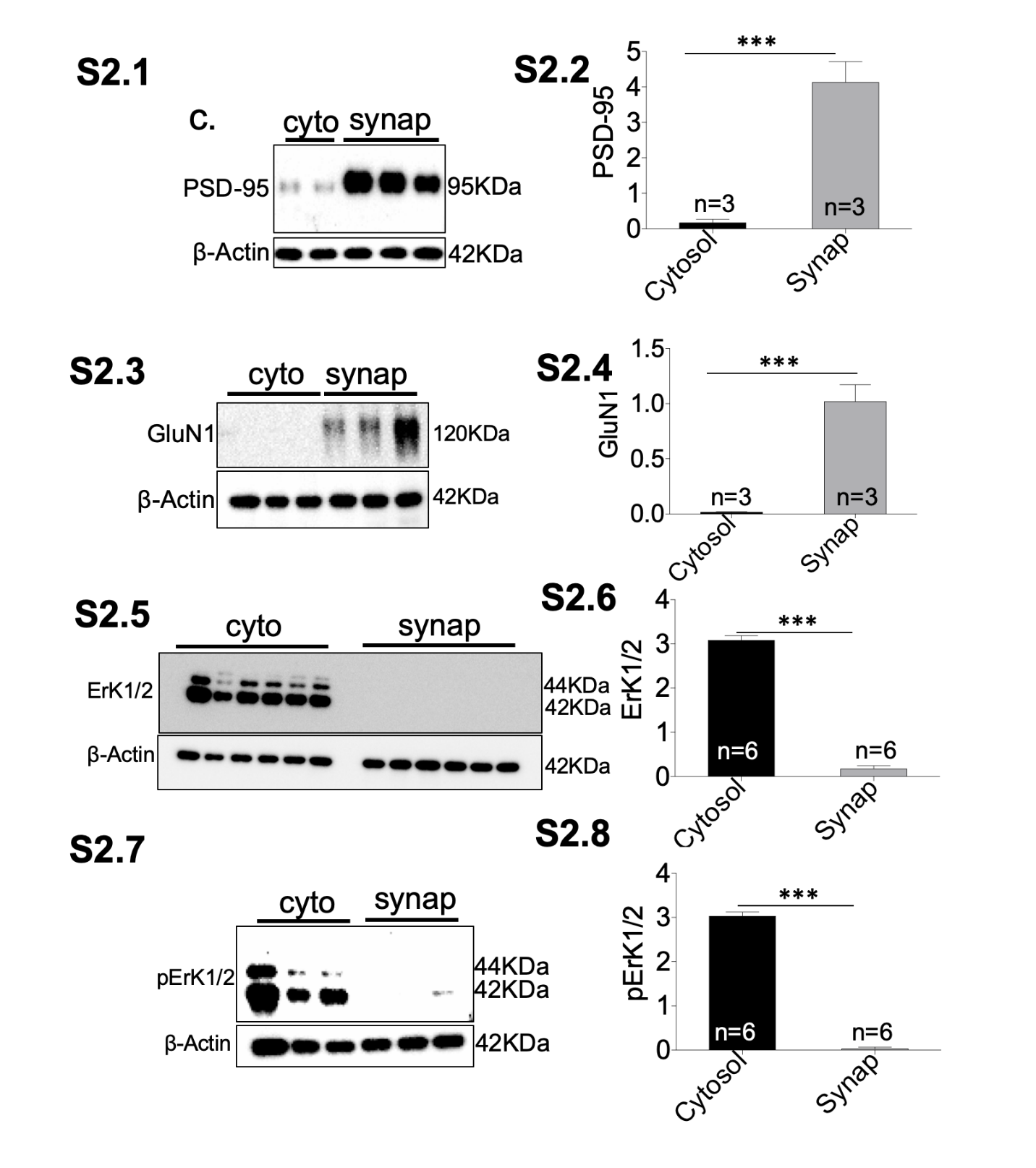


**Figure S2**

Immunoblot validation of synaptosomal and cytosolic extracts isolated by sucrose-gradient technique. PSD-95 (Fig. S2.1-S2.2), GluN1 (S2.3-S2.4), ErK1/2 (S2.5-S2.6), and pErK1/2 (S2.7-S2.8). (***p<0.001).


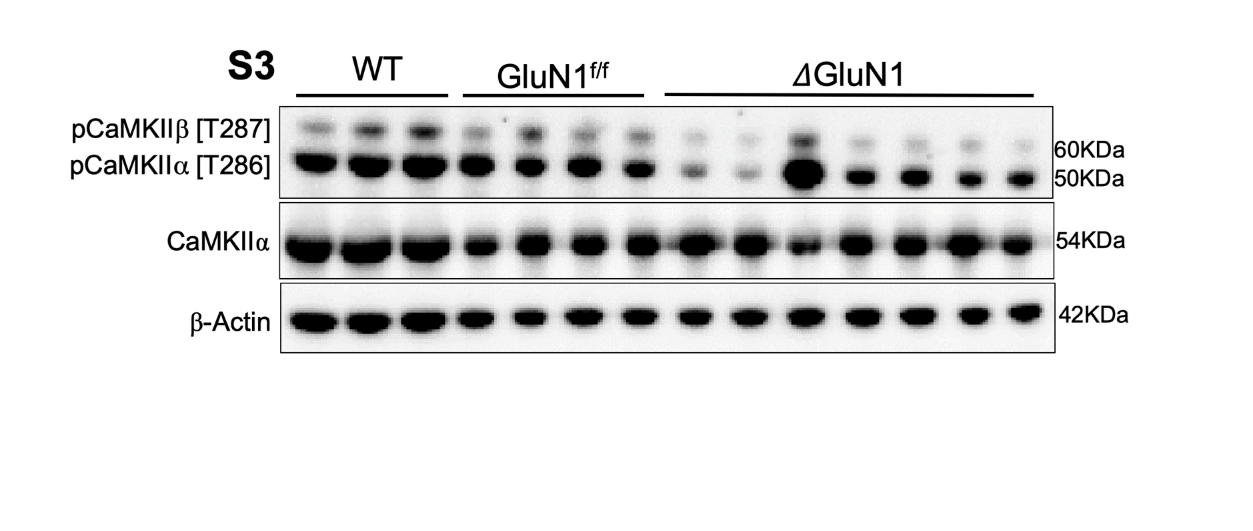


**Figure S3**

Complete immunoblot image for Fig. 6D.


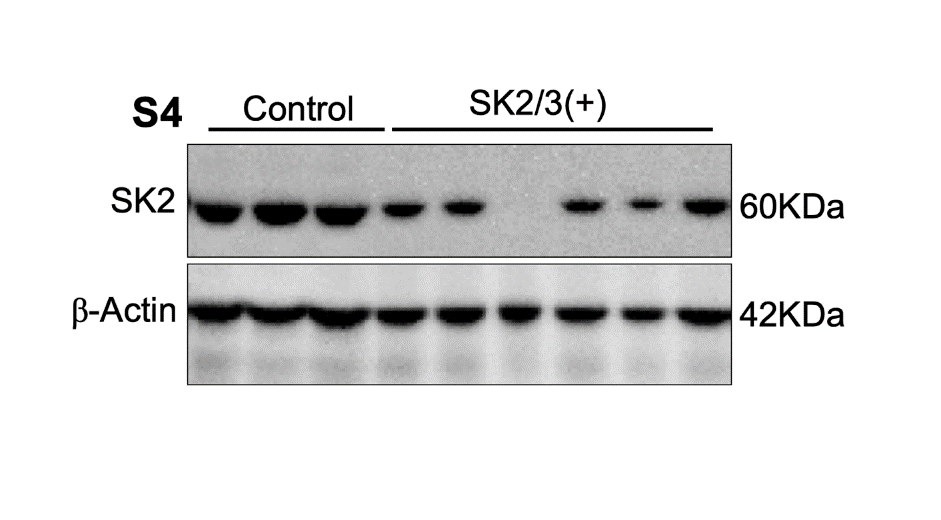


**Figure S4**

Additional immunoblots illustrating the loss of SK2 in the hippocampus of SK2/3 mice (see Fig. 7c-d).


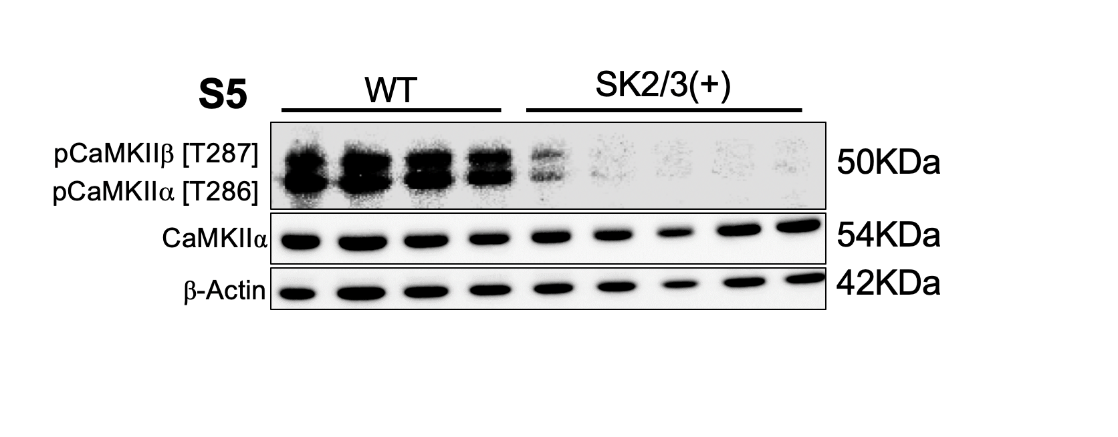


**Figure S5**

Additional immunoblots illustrating the loss of T286 pCaMKIIα in the hippocampus of SK2/3 mice (see Fig. 8a).
